# Geochemical variability as an indicator for large magnitude eruptions in volcanic arcs

**DOI:** 10.1038/s41598-022-19902-1

**Published:** 2022-09-23

**Authors:** Gregor Weber, Tom E. Sheldrake

**Affiliations:** 1 Department of Earth Sciences, University of Oxford, Oxford, OX1 3AN UK; 2 Department of Earth Sciences, University of Geneva, 1205 Geneva, Switzerland

**Keywords:** Geochemistry, Petrology, Volcanology, Natural hazards

## Abstract

Caldera-forming eruptions have the potential to induce drastic socioeconomic change. However, the criteria to identify volcanoes capable of producing large magnitude eruptions in the future are not well constrained. Here we compile and analyse data, revealing that volcanoes which have produced catastrophic caldera-forming eruptions in the past, show larger ranges of erupted magma geochemistry compared to those that have not. This suggests geochemical variability is related to the size of magmatic systems. Using heat transfer simulations, we show that differences in magma flux result in a dependency between chemical diversity and magma volume that is consistent with these observations. We conclude that compositional spread should be included in the catalogue of criteria to identify volcanoes with greater probability of producing future large eruptions. Importantly, this allows to identify stratovolcanoes with caldera-like geochemical signatures, which have not yet been recognized as systems with greater likelihood of producing large magnitude eruptions.

## Introduction

Large magnitude, caldera-forming eruptions, classified with Volcanic Explosivity Index (VEI) of 7, occur on average once or twice every thousand years on Earth^1,2^. Analysis of past large magnitude eruptions provides evidence of considerable environmental impacts, including widespread cooling^3,4^, which resulted in cultural disruption including forced migration and increased mortality^5,6^. In today’s technologically interconnected world, an eruption of this type would likely have cascading impacts on critical infrastructure that go beyond historical accounts, stressing the need to address challenges in forecasting capabilities for such events^7^. Large caldera-forming eruptions have never been monitored with modern geophysical or geodetic techniques, but due to experience with smaller explosive eruptions in the past, short-term warning signals in the form of seismicity, surface deformation, thermal anomalies and increased degassing will likely be detected^8–10^. At present, available geophysical imaging studies have not yet identified a particular volcano with large, interconnected melt volumes in the shallow crust, capable of producing a massive caldera-forming eruption. Although crustal magma bodies have accumulation histories that span hundreds of thousands to millions of years^11–14^, diffusion studies indicate that the final melt mobilisation process may operate over timescales of years to centuries for large and smaller eruptions alike^15–17^. This disparity between accumulation and mobilisation timescales may impede the long-term identification of candidate volcanoes by geophysical means.

To anticipate volcanoes that could produce large magnitude eruptions in the future, Newhall and colleagues^18^ developed and applied six criteria, including high rates of magma supply from the lower to upper crust. Such rates of magma transfer are inherently difficult to assess but recent progress in thermochemical modelling of magmatic systems indicates that the chemical diversity of volcanic rocks provides a record of crustal magma fluxes^19^. Here we further explore this hypothesis, starting from a data perspective and show that larger volcanic systems produce a greater variety of erupted bulk-rock compositions. Finally, we use thermochemical modelling to simulate the trans-crustal evolution of magma plumbing systems and show that such behaviour can be explained by differences in magma accumulation rates.

## Results

### Long-term compositional diversity of arc volcanoes

**Figure 1:**
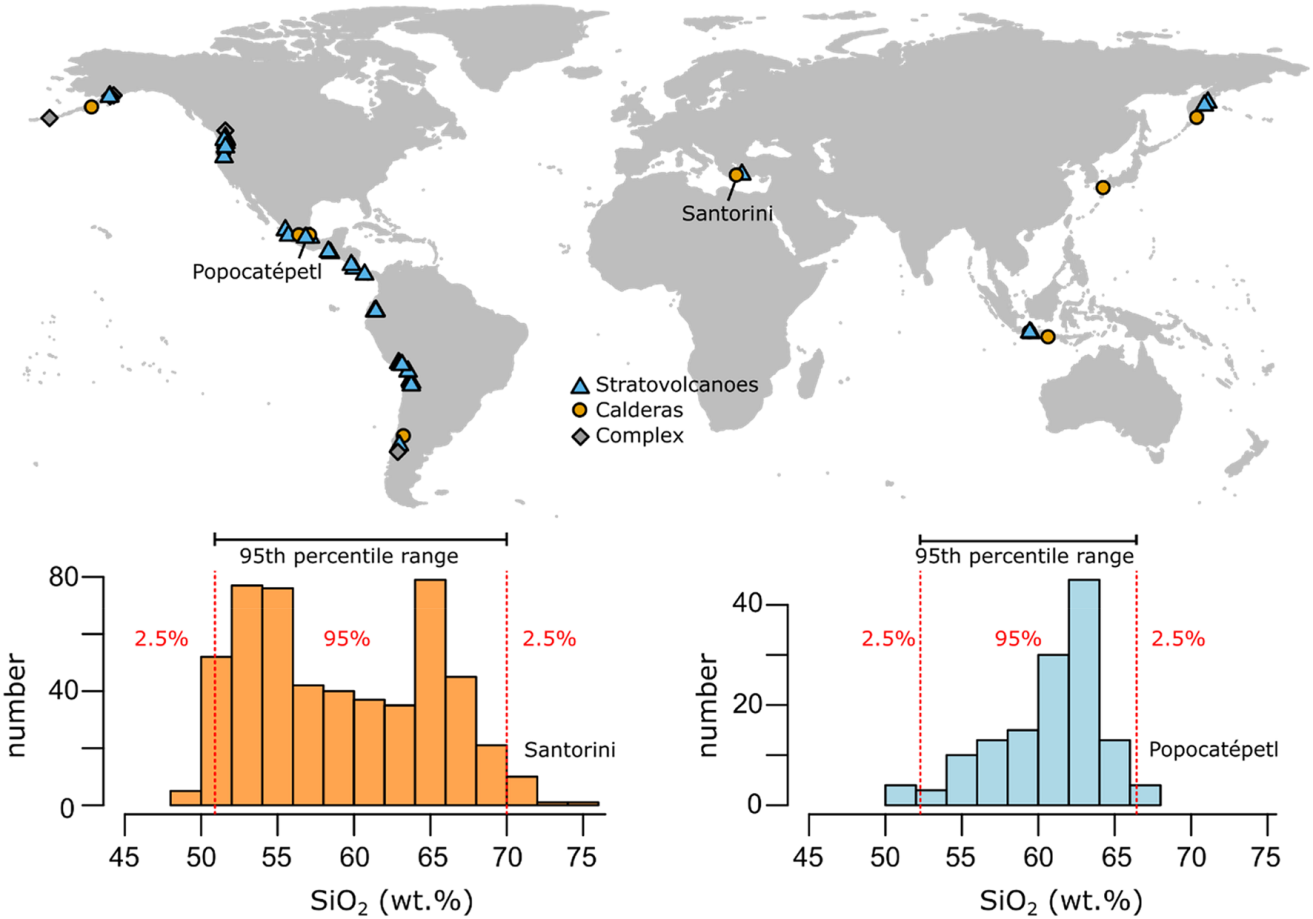
Distribution of the arc volcanoes compiled and analysed in this study. The world map (created using the ggmap package in R) shows the location of stratovolcanoes (blue triangles), calderas (orange circles), and volcanic complexes (grey diamonds). Volcanic bulk-rock analyses were compiled based on the availability of detailed geochemical studies on the long-term eruptive history of individual centres. Histograms of bulk-rock SiO_2_ contents in weight percent are shown for Santorini volcano (Greece, orange-left) and Popocatépetl (Mexico, blue-right) to illustrate the calculation of 95% percentile ranges, cutting off 2.5% of each side of the distributions.

**Figure 2:**
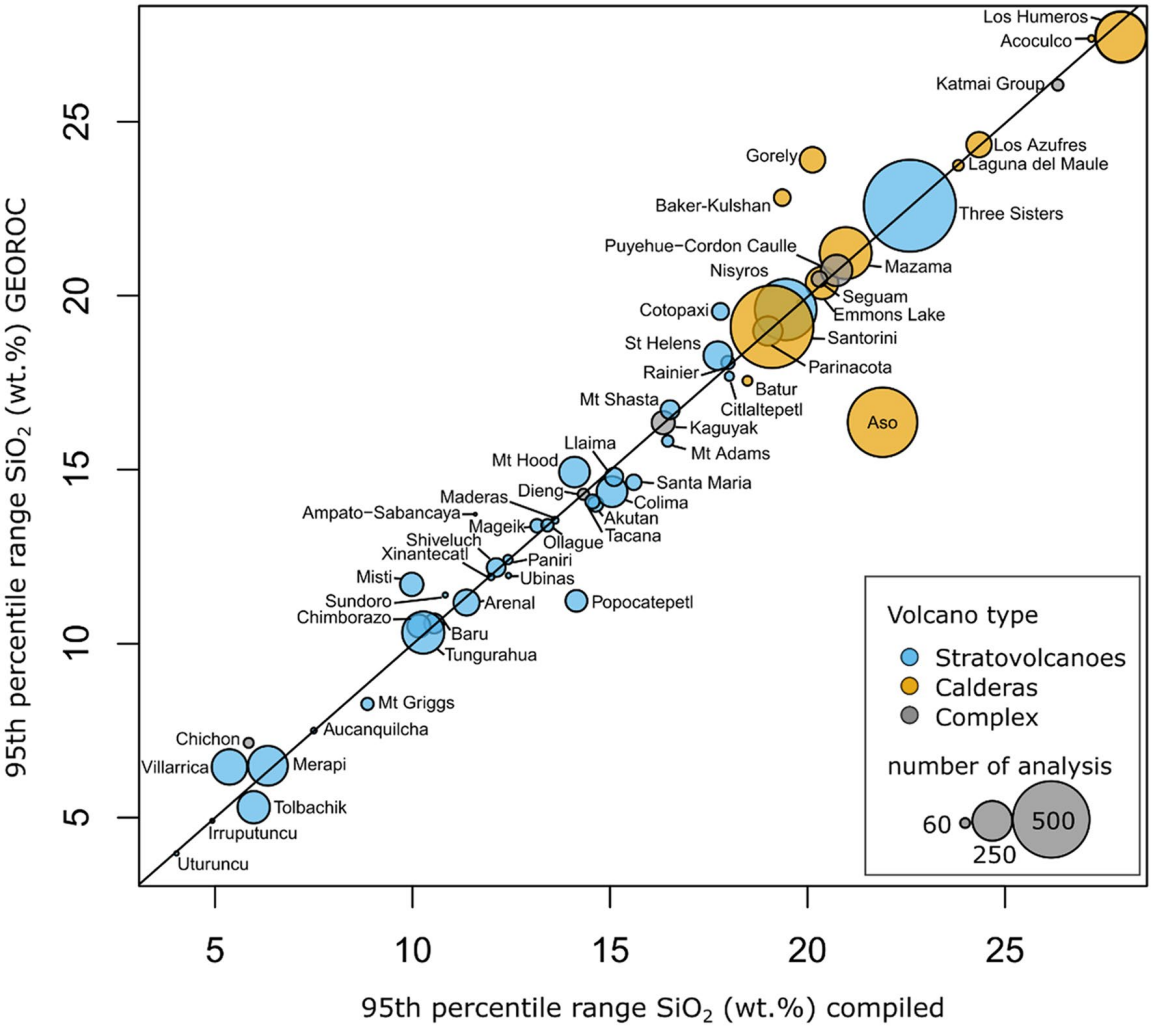
Comparison of erupted bulk-rock SiO_2_ contents in the GEOROC database and compilation. The compiled range of erupted compositions shown as 95th percentile ranges of the SiO_2_ contents (wt%) for each volcanic system is compared to the unfiltered range from the GEOROC database (http://georoc.mpch-mainz.gwdg.de/georoc/). Black line indicates a 1:1 relation. Symbol size is proportional to the number of analysis in the compiled dataset. Colour coding reflects volcano type: Stratovolcanoes (blue), Calderas (orange), Complex (grey).

We identified 54 volcanoes, distributed across 11 arcs, with sufficiently well characterised eruptive histories to quantify their bulk-rock compositional spread (Fig. 1). Within this compilation, stratovolcanoes comprise the largest group with 69%, followed by calderas (20%) and complex volcanoes (11%). To quantify the bulk-rock diversity for each volcano, we calculate the difference between the 97.5th and 2.5th percentile of the distribution of SiO_2_ contents in weight percent, hereafter termed ‘95th percentile range’, which includes 95% of the data by cutting off 2.5% of each side of each volcano’s distribution to reduce sensitivity to outliers. We use SiO_2_ contents as a measure of differentiation, given that they show the largest variation over the igneous spectrum, but also consider other major element oxides (Figs. S1, S2). Elements evolving on curvilinear trends (e. g. MgO, TiO_2_) mostly track source variability in calculated 95th percentile ranges, but oxides showing relatively uniform variation during magma evolution (e. g. CaO, K_2_O, FeO) behave analogous to SiO_2_ when considering 95th percentile ranges of erupted compositions. The usage of different measures of compositional variability, like standard deviation or inter quartile range, does not substantially alter the structure of the data (Figs. S3, S4; Data Repository). Comparing the 95th percentile range of SiO_2_ contents for each volcano in our compilation to those calculated for data from the GEOROC database, most volcanic systems fall close to a 1:1 line (Fig. 2). Notable differences between the compilation and the GEOROC database can be explained by the overrepresentation of the prominent eruptions, and data of unclear provenance that has been removed while compiling data. For example, the higher range of SiO_2_ contents in the compiled dataset (21.9 wt% SiO_2_) compared to GEOROC (16.4 wt% SiO_2_) for Aso volcano in Japan result from exclusion of studies only focussed on the Aso-4 eruption, the largest and most studied eruption from this location. It should be noted that the number of analysed samples does not correlate with the range of erupted bulk-rock compositions (Fig. 2), showing that the sample size is not a primary control on the compositional variability of volcanoes in the dataset. Volcanic systems with a small number of bulk-rock analysis (e. g. n = 42 for Acoculco, n = 68 at Laguna del Maule) can show large compositional spread, while centres such as Merapi (n = 251) or Tungurahua (n = 266) maintain their monotonous chemistry even at large quantities of analysed samples.

**Figure 3:**
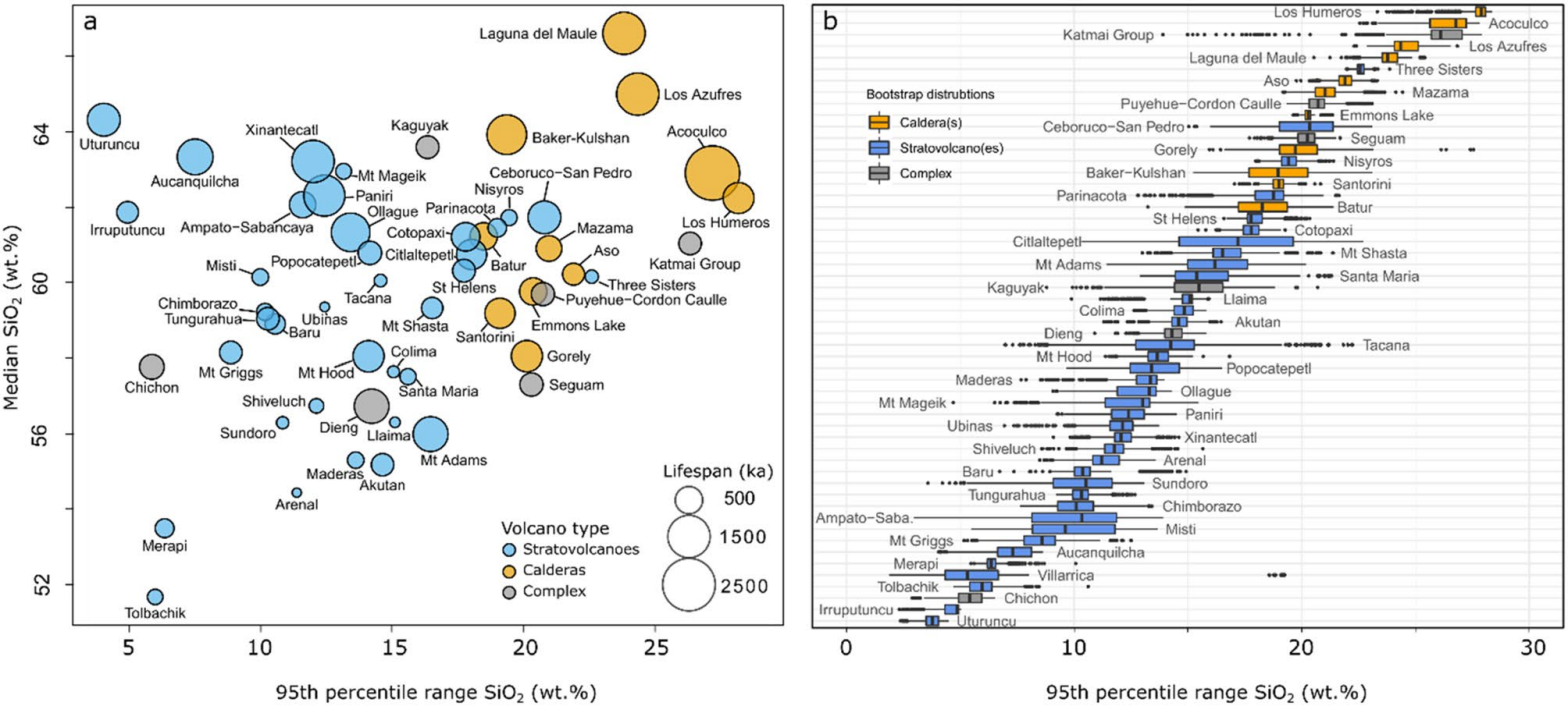
Spread of erupted SiO_2_ bulk-rock contents (wt%) for different volcano types. (**a**) Median SiO_2_ content (wt%) is plotted versus the 95th percentile range of erupted bulk-rock compositions. Different volcano types are indicated by colour: orange (calderas), blue (stratovolcanoes), grey (complex systems). The symbol size reflects the total duration of activity for each volcanic system as constrained by radiometric dating (Supplementary electronic dataset 2). (**b**) Bootstrap distributions of the 95th percentile range for the volcanic systems. The data for each volcano were resampled (‘bootstrapped’) 3000 times with replacement according to the original sample size. Boxplots show the spread of resampled distributions for each volcano and were ranked by the median.

The erupted whole-rock SiO_2_ distributions show large differences in the 95th percentile range of erupted compositions and reveal systematic differences between volcano types (Fig. 3). SiO_2_ 95th percentile ranges in our compilation vary between 4 wt% for Uturuncu volcano in the Bolivian Altiplano and 28 wt% for the Los Humeros caldera in in the Trans Mexican Volcanic Belt, with most systems recording intermediary ranges of 10–20 wt%. Median SiO_2_ contents shown in Fig. 3a to illustrate the overall degree of differentiation of the various volcanic systems in the compilation relative to each other, spanning the entire range from dominantly basaltic and intermediate centres such as Tolbachik (Kamchatka, Russia) and Colima (Trans Mexican Volcanic Belt), to rhyolitic calderas like Laguna del Maule (Southern Volcanic Zone, Chile). Although most volcanoes in our dataset are likely fed by mantle derived basalts, such magmas are not necessarily erupted at all systems, leading to monotonous silicic volcanoes such as Uturuncu in Bolivia or Xinantécatl in the Mexican Volcanic Belt. The 95th percentile range of SiO_2_ contents of volcanoes is therefore not simply a measure of the difference between the most evolved composition and basaltic input magma but reflects more complex sampling behaviour.

The most important observation from Fig. 3 is that caldera volcanoes clearly show a systematically higher spread in erupted bulk magma chemistry compared to the great majority of stratovolcanoes. Volcanic systems that have produced caldera-forming eruptions vary in their 95th percentile ranges of SiO_2_ contents between 19 and 28 wt% with median 95th percentile range of 21 wt%, while stratovolcano 95th percentile ranges show a large spread between 4 and 23 wt% but most systems show less geochemical diversity compared to calderas, as indicated by the overall median for stratovolcano 95th percentile ranges of 13 wt%. To assess the impact of the variable sample size, calculated 95th percentile ranges of the SiO_2_ contents in weight percent were randomly sampled 3000 times with replacement (i.e. compositions can be sampled multiple times from the dataset) according to the original sample size for each volcanic system. By this resampling procedure, also called ‘bootstrapping’, a confidence interval for the distributions of 95th percentile ranges can be established (Fig. 3b). Clearly, the resampled distributions of bulk-rock geochemical diversity differ as a function of volcano type given 90% confidence levels of 17–28 wt% SiO_2_ for calderas and 5–20 wt% for stratovolcanoes. Using a Brown-Forsythe test (R package: ‘onewaytests’) for the equality of group variances on the data presented in supplementary Table S1, we show that erupted bulk-rock SiO_2_ 95th percentile ranges differ significantly among stratovolcanoes and calderas with *p*-value of 1.6025e−07 (1 degree of freedom, F-statistic: 55.65), supporting our result that caldera volcanoes show systematically higher ranges in erupted chemistry. Interestingly, several stratovolcanoes like Three Sisters (Cascades) or Ceboruco (Trans Mexican Volcanic Belt) have erupted compositional distributions, which are more akin to those of calderas rather than other stratovolcano in the compilation.

### Potential impact of caldera-formation on erupted magma variety

**Figure 4:**
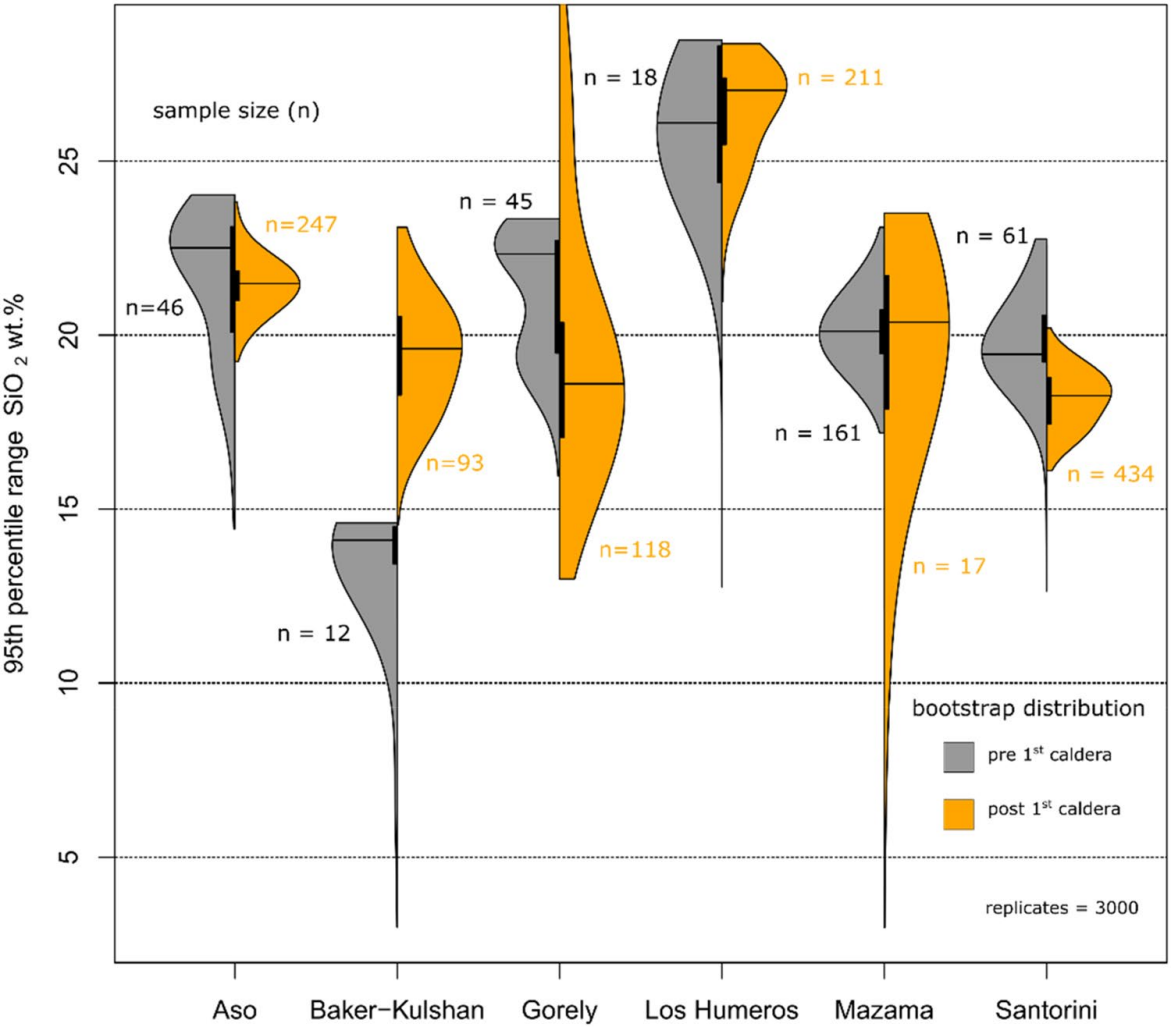
Spread of erupted bulk-rock geochemistry in relation to caldera-formation. Density distributions of the 95th percentile range were calculated by resampling compositions before the first caldera forming eruption (pre 1st caldera, grey colour) and after the first caldera formation (post 1st caldera, orange colour) 3000 times according to original sample size (n). The interquartile range (vertical black bar) and median (horizontal black line) of each density curve are also shown. Five out of six caldera systems show no difference in the spread of erupted magma chemistry before and after the first caldera-forming eruption, showing that caldera-formation itself is not the primary factor that created the large compositional spread of these systems.

The observation that caldera volcanoes show larger spread of erupted bulk-rock geochemistry may either follow from internal properties of their magmatic feeding systems (e. g. crustal magma injection rate) or could be a consequence of the structural collapse itself, resulting from explosive caldera-formation. The latter process may be associated with a change in the crustal stress field^20^, potentially modifying the capacity of the volcanic system to sample a greater variety of geochemical compositions from a larger depth range. As the question if geochemical diversity of caldera volcanoes is primarily a consequence of internal (i.e. magmatic) or external (i.e. structural) processes impacts on the applicability of our finding (discussed below), we test this hypothesis by analysing data before and after the first caldera-forming eruption for different volcanoes. Of the 11 calderas in our compilation, six volcanoes (Aso, Baker-Kulshan, Gorely, Los Humeros, Mazama, Santorini) have enough data available to quantify the bulk-rock diversity before and after the first caldera-forming event. As shown in supplementary Table S1, no data of the pre-caldera phase are available for Emmons Lake (Alaska), Laguna del Maule (Chile), Los Azufres (Mexico), and only limited data could be obtained for Batur (Indonesia, n = 4 whole-rock analysis) and Acoculco caldera (Mexico, n = 2). Consequently, these systems were discarded from this part of the analysis. Detailed notes on sample inclusion of the pre- and post-1st caldera phases, as well as histograms of the raw data (Fig. S3) for each of the analysed volcanoes are presented in the supplementary materials to this article. To establish confidence intervals for the magma diversity of pre-caldera and post-1st caldera phases, we resampled the distributions of SiO_2_ contents 3000 times with replacement for each caldera volcano. As shown in Fig. 4, even though the pre-caldera phase has lower preservation potential, younger units may cover it, and is typically less well-studied, five out of six caldera volcanoes show equal or larger 95th percentile ranges prior to the first caldera-forming event. Histograms of the raw bulk-rock data are presented in the supporting materials, including detailed notes on data compilation for the different caldera stages for each considered volcano. An exception to this behaviour represents the Mt Baker-Kulshan caldera system (Cascades), which shows lower magma diversity in the pre-collapse stage. However, it is unclear whether this distribution at Mt Baker-Kulshan caldera represent the true variability of erupted rocks in the pre-caldera stage, in particular as the area has been highly impacted by glacial erosion, which has removed the entire outflow ignimbrite sheet and likely pre-caldera rocks as well^21,22^. In either case, the great majority of the available data shows no difference in the transition from pre- to post-1st caldera volcanism, making the hypothesis that the large compositional spread of calderas is purely a result of collapse processes and associated changes in stress field untenable.

### Spatial analysis of volcanic rock diversity

**Figure 5:**
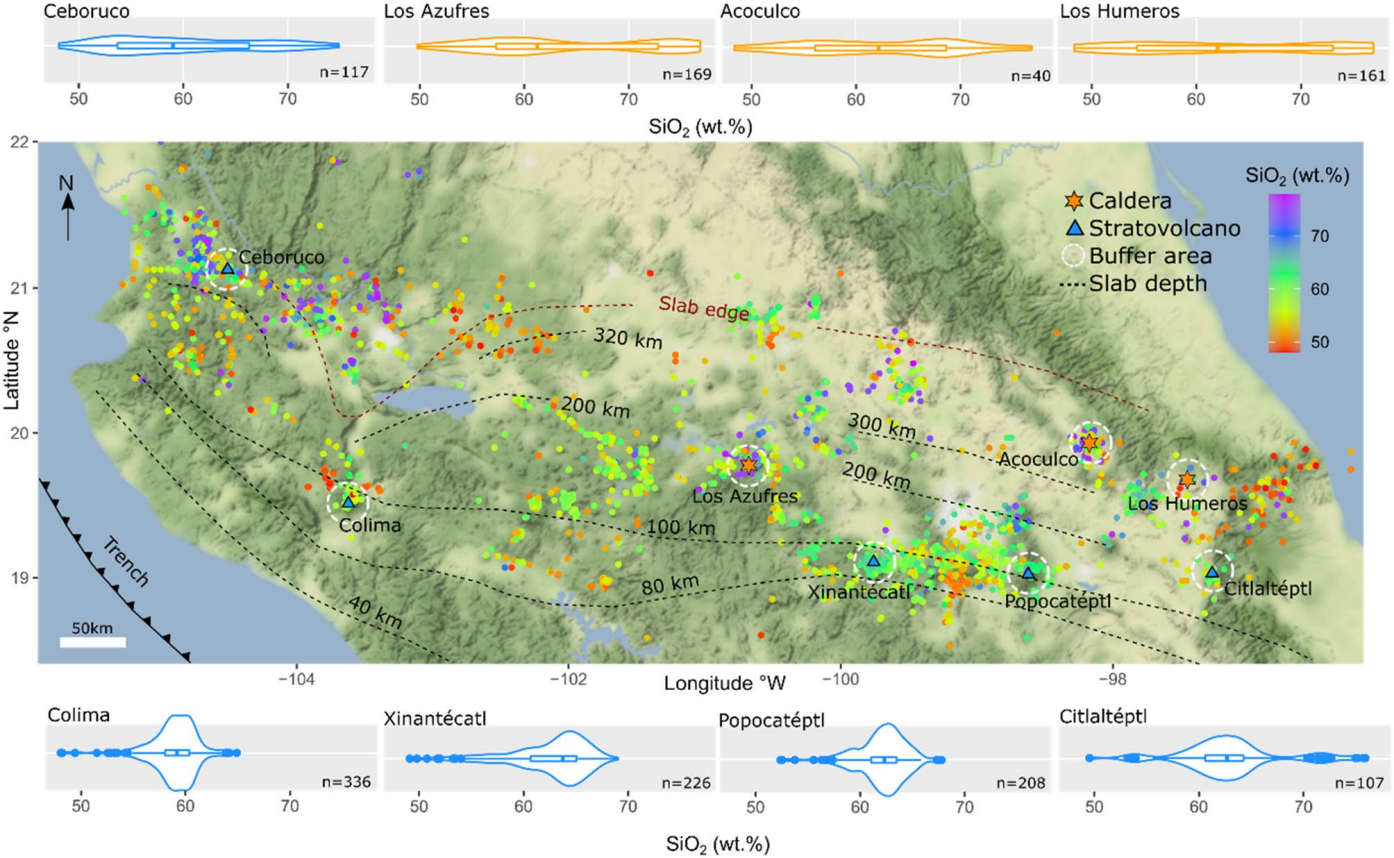
Spatial distribution of SiO_2_ contents (wt%) in bulk-volcanic rock analysis of the Trans Mexican Volcanic Belt. Points on the map show the location of bulk-rock analysis from the GEOROC database, colour coded for their SiO_2_ content. The location of calderas is marked by orange stars, stratovolcanoes are shown by blue triangles. Only volcanoes with constrained eruptive history are shown. Dashed circles (30 km buffer area) surrounding the volcanoes indicate the area, in which analyses were included to calculate the compositional distributions. Slab depths were taken from reference^23^. Colour of the density distributions reflects volcano type (stratovolcano: blue; caldera: orange). The map was created using the ggpmap package in R. Terrain Map tiles by Stamen Design, under CC BY 3.0., and map data by OpenStreetMap, under ODbL.

Before discussing underlying mechanisms, limitations, and implications our finding that caldera volcanoes are geochemically distinct from most stratovolcanoes, we turn to the evaluation of how the observed differences may be impacted by spatial sampling effects. Calderas typically comprise a larger spatial area compared to most stratovolcanoes, which could potentially bias the results due to differences in the extent of sampling. It is therefore pertinent to compare the spread of bulk-rock geochemistry for stratovolcanoes and calderas based on samples within equal areas. We use the Mexican Volcanic Belt as a test case to visualize and compare the spatial distribution of erupted compositions, given that it contains a comparably large number of volcanoes in our compilation (Fig. 5). Compositional distributions for eight volcanoes were calculated by including all available data of the GEOROC database within equal ‘buffer areas’ of 30 km diameter surrounding the main vent. As shown in Fig. 5, the differences in shape of the resulting distributions based on spatial sampling confirm our results obtained by analysis based on a volcanic system approach (Figs. 2, 3). All calderas in the Mexican Volcanic Belt (i.e. Los Humeros, Acoculco, and Los Azufres) show broader SiO_2_ distributions compared to four out of five stratovolcanoes, which typically show more confined distributions. Again, Ceboruco volcano shows a geochemical distribution akin to those of caldera volcanoes, which may relate to fundamental differences in the architecture or dynamics of its magmatic systems compared to other Mexican stratovolcanoes.

## Discussion

**Figure 6:**
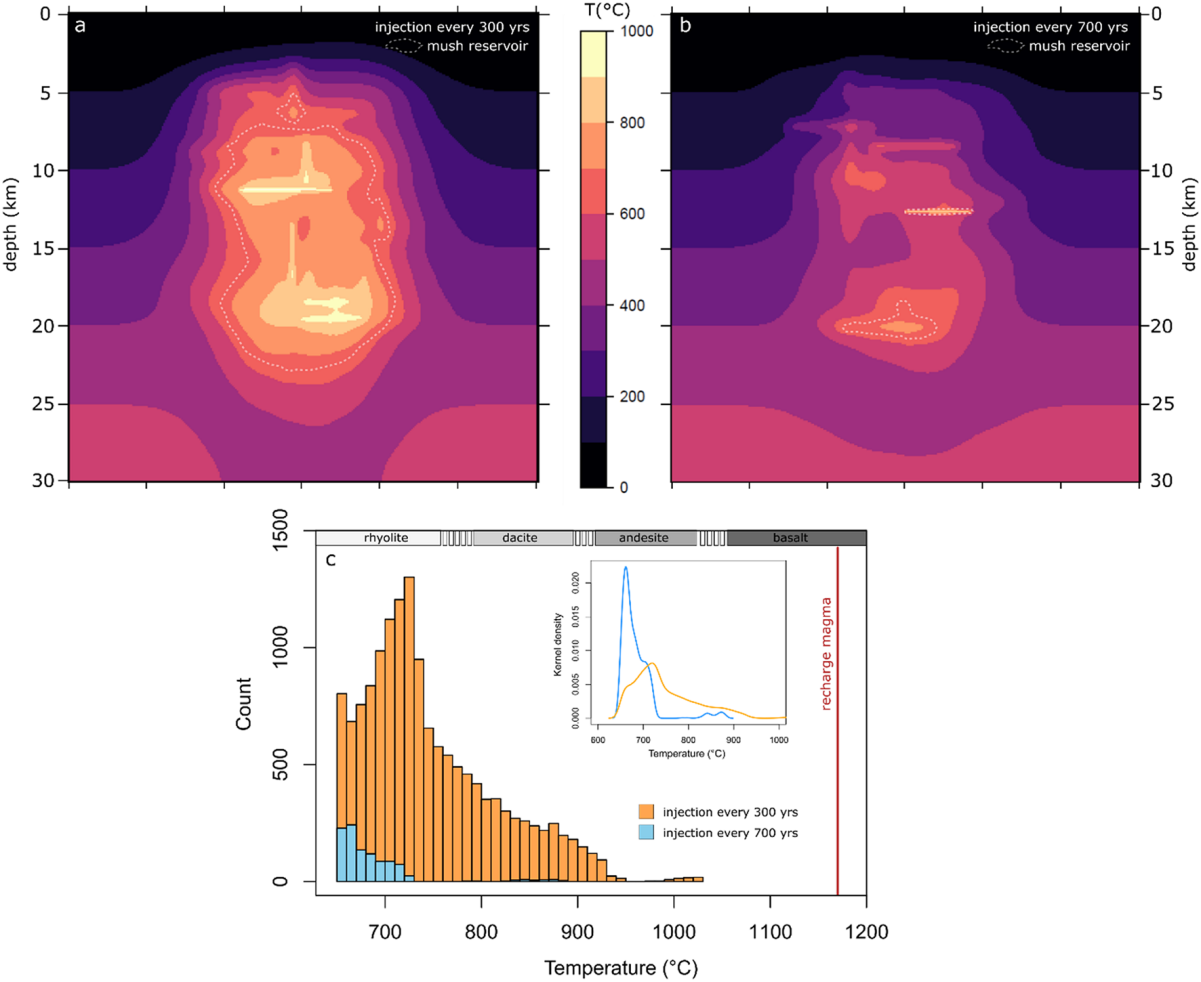
Relationship of compositional diversity and thermal state of igneous plumbing systems. (**a**) Two-dimensional temperature (°C) cross section through a crustal magmatic system built by injection of dikes and sills at a pulse rate of one injection every 300 years for 100,000 years. Dikes and sills were randomly injected over the depth range of 4 to 20 km with basaltic composition at liquidus temperature of 1170 °C. (**b**) Crustal plumbing system built by the same parameters as in (**a**) but at lower magma injection rate of one pulse every 700 years, resulting in less voluminous mush bodies and smaller temperature range of magma in the plumbing system. (**c**) Histogram of temperatures for scenario (**a**) is shown in orange and (**b**) in blue. The recharge magma composition is indicated by a vertical red line and approximate temperature ranges of magma compositions are shown. Inset shows the kernel density distribution of the histogram.

Caldera-forming eruptions of VEI-7 require the accumulation of magma volumes sufficient to produce > 100 km^3^ of tephra, suggesting that the conditions favouring this also result in wide ranges of erupted bulk-rock compositions. Due to the coupling between melt temperature and chemistry, which has been shown in numerous experimental studies^24–29^, we propose that geochemical variability is related to thermal heterogeneity in spatially extensive magmatic plumbing systems^19^. We suggest the primary factor favouring compositional variability and larger volumes of silicic melt is higher magma injection rates, which is shown using thermal modelling of stochastic magma injection and evolution in the crust (Fig. 6, see methods section). For simplicity, we do not consider the ability to accumulate or erupt magma in the numerical modelling, although this will certainly determine the ability of a volcano to produce large-magnitude eruptions with VEI of 7. Nevertheless, it has been shown that without high magma fluxes, it is impossible to accumulate large volumes of magma^30^, and so determine this to be the fundamental control on magma composition diversity. Over equivalent total durations of basaltic magma injection, higher magma injection rates (e. g. one pulse every 300 years) into the crust leads to a greater vertical extent of the plumbing system and larger silicic mush volumes (Fig. 6a) compared to lower magma flux cases (Fig. 6b, e.g. one pulse every 700 years). Such behaviour will inevitably result in a greater distribution of different temperatures for the higher injection rate scenarios and more monotonous thermal structure in the low magma flux case (Fig. 6c). Heat conduction and convective stirring, as well as latent heat buffering may also lead to the build-up of monotonous magma bodies over protracted episodes of time that have been documented at the level of individual large magnitude (often VEI-8) eruptions^31,32^. However, the observation that more voluminous volcanic systems show greater ranges of erupted compositions (Fig. 3) is consistent with larger thermal variability in more extensive crustal feeding systems as shown through thermal modelling (Fig. 6).

Whilst magmatic systems may experience geochemical variability with time^21,33–40^, we suggest at the global scale the maturity of a volcano is less significant than magma flux, as the lifespan of volcanoes is not correlated to smaller or larger geochemical variability. As shown in Fig. 3a, caldera systems producing diverse magma chemistry like Mazama, Santorini, or Los Humeros have lifespans equivalent or shorter than many examples of long-lived stratovolcanoes (e. g., Uturuncu, Aucanquilcha, Xinantécatl) that have produced monotonous andesitic-dacitic magma chemistry throughout their > 1.5 Ma histories^41–43^. In order to maintain their monotonous character over such long timescales, pre-eruptive magma storage depth and temperatures for such volcanoes must be relatively constant and balanced by eruption rates^44^. For younger, more monotonous basaltic systems (e.g., Tolbachik, Merapi; Fig. 3) we cannot rule out that these volcanoes may evolve to larger more heterogenous magmatic systems over time. However, the frequent eruption record at these volcanoes^45,46^ and implied low-residence time of magmas in the upper crust, suggests that conditions do not favor the development of spatially extensive reservoirs. Nevertheless, some of the longest-lived caldera volcanoes exhibit high geochemical variability, which is consistent with progressive thermo-mechanical softening of crustal rocks^47–49^, suggesting the maturity of a magmatic system (i.e. volcano age) can still be an important factor to accumulate large volumes of eruptible magma. A causal relationship between the spread of erupted magma geochemistry and the temporal transition from the pre- to post-1st caldera-forming eruption stage is not evident based on our analysis.

The development of caldera systems results from the complex interplay of tectonic and crustal processes that influence the ability to accumulate large magma volumes^50,51^. The regional impact of these processes is observed in the Trans Mexican Volcanic Belt. Here the active volcanic front is dominated by similar low-variability magmas, whereas further from the active front volcanic centres produce a diverse array of magma geochemistry (Fig. 5). An identical pattern is observed in the type of volcanoes, with the active front characterised by andesitic stratovolcanoes in comparison to large calderas^52^. One exception is Ceboruco, which has not produced a large caldera, but lies behind the active front. Nevertheless, Ceboruco exhibits large geochemical variability (Figs. 3, 4). We suggest this variability results from the same processes that favour the accumulation of magma feeding the large caldera-forming eruptions at other systems (Fig. 4). Such close spatial association of compositionally monotonous and highly variable volcanic systems emphasises the important role of local differences in crustal magma fluxes^13,19,53–55^.

**Figure 7:**
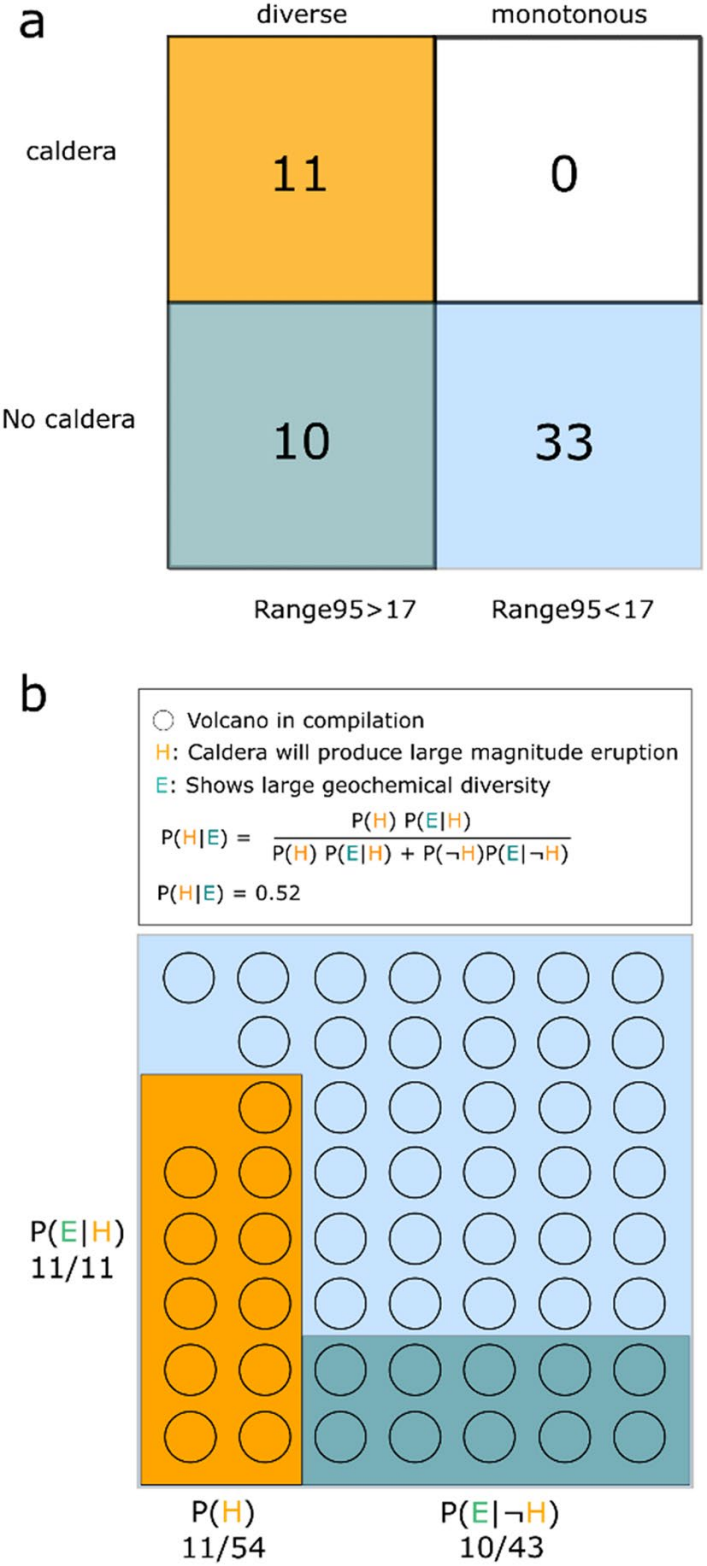
Contingency table and Bayesian inference for volcanoes in the compilation. (**a**) Volcanic systems were categorized as monotonous if they showed a 95th percentile range of the SiO_2_ (wt%) distribution of < 17 wt% and as diverse with 95th percentile range > 17 wt% SiO_2_. Further, the compiled volcanoes were categorized into caldera (> 8 km diameter) and no-caldera systems. Orange colour shows diverse caldera systems (n = 11), white: monotonous calderas (n = 0), green: diverse non-caldera (n = 10), and blue monotonous non-caldera (n = 33). (**b**) Each volcano in the dataset is represented by an open circle and by colour coding as in a). The proposition H is the initial belief that a caldera volcano will produce the next large magnitude eruption and B is the observational evidence that a volcano shows large compositional spread. Using Bayes’ theorem, the posterior P(H|E) is calculated, which is the probability that a caldera will produce the next large magnitude eruptions under the belief that geochemical diversity reflects the likelihood of large magnitude eruptions. Hence the alternative, P(H|E), meaning that a compositionally diverse non-caldera volcano will produce the next large magnitude eruption, is inferred with in a probability of 48%.

The relationship between magma diversity and igneous plumbing system size, as indicated by our analysis and modelling, can be used as an indicator of hazardous volcanoes with greater likelihood of producing caldera-forming eruptions. This is important in the pre-caldera phase, especially for stratovolcanoes that can transition to large-magnitude caldera-forming eruptions but have not experienced such events previously. Several stratovolcanoes in our compilation (i. e., Three Sisters, Ceboruco, Parinacota, Nisyros, St Helens) show geochemical distributions akin to those of calderas, indicative of larger plumbing systems size, but these systems have previously not been recognized as capable of producing VEI-7 eruptions based on the criteria laid out in^18^. Although measures of the maximum SiO_2_ content of eruptive products yield promising results in distinguishing caldera and non-caldera systems (Fig. S4), using the range of compositions erupted adds a constraint that separates systems typically producing VEI-7 eruptions and monotonous systems erupting silicic compositions (Fig. 3). We therefore suggest that the compositional spread of volcanoes is included in the list of criteria to evaluate the long-term risk stemming from different volcanoes worldwide. For the arc volcanoes that we have analysed here, assuming all volcanoes that exhibit large variability in their whole-rock geochemistry can produce large magnitude caldera-forming eruptions, then the probability that the next such event will occur at a volcano that has not previously produced a VEI-7 event is ~ 50% (Fig. 7). This estimate assumes that (1) the fractions of non-caldera to caldera systems and that (2) the fraction of compositionally diverse to monotonous volcanoes reflects the global population of arc volcanoes. While the second assumption can only be improved through further studies constraining the compositional diversity of volcanoes, the first assumption can be cross validated against existing compilations. We therefore provide additional estimates based on the Large Magnitude Volcanic Eruptions^56^ (LaMEVE) and Global Volcanism Programme^57^ (GVP) databases. The fraction of volcanic systems classified as caldera in the LaMEVE database is 0.224, similar to our compilation value of 0.204, resulting in a slightly lower probability of 45%. Using the combined Pleistocene and Holocene volcano list from GVP, a lower caldera fraction of 0.09 is calculated, yielding a probability of 70% for a VEI-7 eruption to be produced by a geochemically diverse non-caldera volcano. In either case, given the overall tendency to focus monitoring efforts on volcanoes that have previously produced large magnitude eruptions, our analysis demonstrates the need to identify and use criteria in the search for high-risk volcanoes that are independent of the volumes of previous eruptions. As the eruptive history of most volcanoes worldwide is unconstrained, further studies are needed aiming to establish the record and diversity of volcanic rocks.

## Methods

### Data compilation

Accurately quantifying the bulk-rock chemical spread of volcanic systems requires that: (1) the long-term eruptive history is well resolved; and (2) biases are identified, evaluated and, if problematic, eliminated. Given the large range of sampling strategies in geochemical studies and differences in the extent to which eruptive histories of volcanoes can and have been reconstructed, we argue that no single set of filtering criteria (e.g. total number of analysed samples or spatial extent of sampling) for existing large geochemical databases should be utilized to quantify the spread in bulk-rock geochemistry of volcanoes. Therefore, we carefully compiled data, using the GEOROC database as a starting point, which was complemented by literature search. In the GEOROC database, all precompiled files by location ’Convergent Margin’ were considered. Volcanoes were only included in the compilation with at least one published study, specifically targeting the long-term geochemical evolution of the system as constrained by geochronology. If that condition applied, all available references presenting geochemical data for the volcano were examined to remove detailed studies on a particular eruption and oversampling of prominent eruptions, which could potentially bias the compositional spread towards lower values. We note, however, that both potential biases are rare based on available data. Further, data inclusion in the compilation was based on two main criteria: (1) Spatial association to the volcanic system within reasonable distance from the central vent (typically < 20 km). (2) Eruptive time series without significant gaps of several hundred thousand years to millions of years. Thus, studies with unclear spatial or temporal relation to the volcanic centre under investigation were removed. To be conservative in data inclusion, no filters based on analytical totals or loss on ignition were applied, as the risk of introducing biases for different rock groups this way outweighs slight differences in overall compositional spread resulting from analytical totals. However, to restrict the compositional range to typical basaltic to rhyolitic compositions we included only analyses within a SiO_2_ contents between 48 and 78 wt%. We focus only on arc volcanoes to avoid problems of intercomparison between different tectonic settings. This is necessary as contrasting endmembers of magma differentiation (i. e. basanite-phonolite, basalt-rhyolite), will lead to differences which are problematic to describe with the same index of differentiation and measure of compositional spread. The primary volcano type was assigned for each system based on the Smithsonian Holocene volcano list (https://volcano.si.edu/list_volcano_holocene.cfm) and calderas dataset of Hughes and Mahood (reference^50^). As individual volcanic centres may be comprised of a multitude of landforms, we used an objective criterion, which is the presence or absence of a collapse cauldron with diameter > 8 km to distinguish calderas from stratovolcanoes, and complex volcanoes, including larger dome complexes and composite systems for which a unique classification is not meaningful. We further assigned volcano lifespans based on geochronological data (Electronic supplementary dataset) and the magnitude of the largest eruption taken from either the LAMEVE database or a literature search for systems where data is available. The full data compilation used in this study is available in the electronic supplementary materials to this article.

### Thermal modelling

We use heat conduction theory to simulate the temporal evolution of temperatures in the Earth crust experiencing injection of magmatic sills and dikes^11,18^. The heat conduction equation in two dimensions is given by:

1 $$\rho c\frac{\partial T}{{\partial t}} = \frac{\partial }{\partial x}\left( {k\frac{\partial T}{{\partial x}}} \right) + \frac{\partial }{\partial z}\left( {k\frac{\partial T}{{\partial z}}} \right) + L_{c} ,$$

where T is the temperature, t is time, rho is the density of 2700 kg m^−3^, c is the specific heat of 1 kJ kg^−1^ K^−1^, k is the thermal conductivity of 2.7 W m^−1^ K^−1^ and L_c_ is the latent heat of fusion of 350 kJ kg^−1^. The equation was discretized on a numerical grid using a fully explicit second order finite-difference scheme^11^. Timestep and spatial resolution were chosen by convergence test. In all simulations we choose an initial linear geotherm of 20 °C/km. Zero flux boundary conditions were applied at all sides except the surface which was set to 0 °C. To include latent heat release, the non-linear melt fraction (M_f_) temperature relation based on fractional crystallisation experiments of Nandedkar et al.^28^ was implemented in the model. Latent heat release was balanced using the heat capacity method and a non-linear iteration loop. Magma batches were injected as 100 m thick vertical dikes through modification of the temperature field, randomly varying in length between 0.5 and 2 km and horizontal sills of 2 to 3 km length. Injection sites were randomized within a subdomain of 4 to 20 km depth and 10 km horizontal extent of the total 30 × 30 km model. Injected magma batches were accommodated by advection of crustal rocks to the sides in case of dikes and downwards for sills.

## Supplementary Information

Supplementary Information 1.

Supplementary Information 2.

## Data Availability

The compiled data used in this study are available in the electronic supplementary materials.
