# Supplementary material for: Geochemical variability as an indicator for large magnitude eruptions in volcanic arcs
Source: Sci Rep. 2022 Sep 23;12:15854. doi: 10.1038/s41598-022-19902-1 (PMC9508169; doi:10.1038/s41598-022-19902-1)
Supplement: Supplementary file 2 — Supplementary Information 2. [file 41598_2022_19902_MOESM2_ESM.pdf]

Supplementary materials

Geochemical variability as an indicator for large magnitude eruptions in volcanic arcs

Gregor Weber[1], Tom E. Sheldrake[2]

[1] Department of Earth Sciences, University of Oxford, United Kingdom

[2] Department of Earth Sciences, University of Geneva, Switzerland

Correspondence: gregor_weber@gmx.de

Contents of this file

Figures S1 to S7

Table S1

Notes on data compilation

Supplementary references

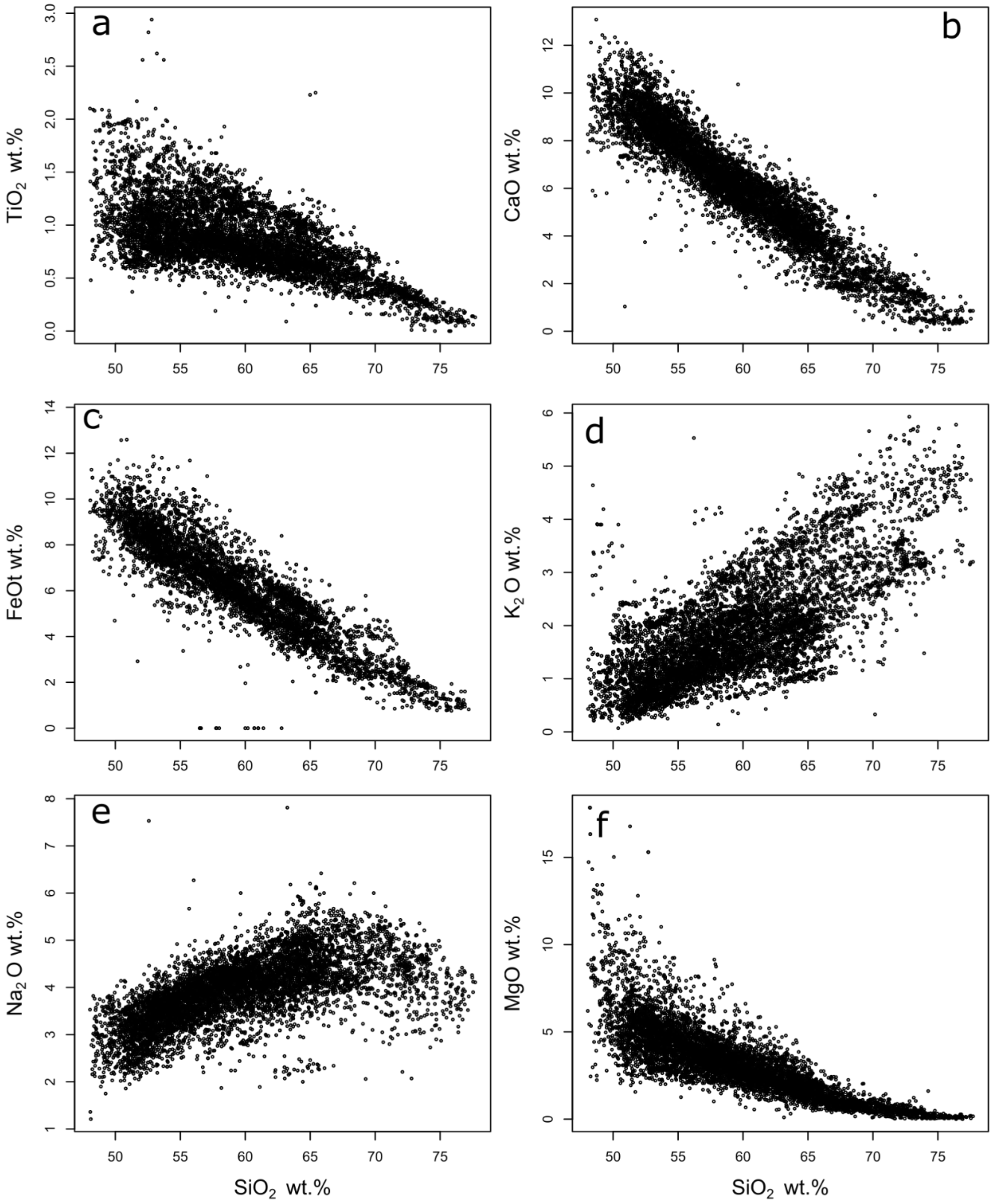

Fig S1: Major element oxides plotted versus SiO_2 in weight percent in the compiled dataset. Note that some major element oxides show a large variability over a restricted range of SiO_2 contents (e.g. MgO, TiO_2), while others show more uniform behaviour (e.g. CaO, FeOt).

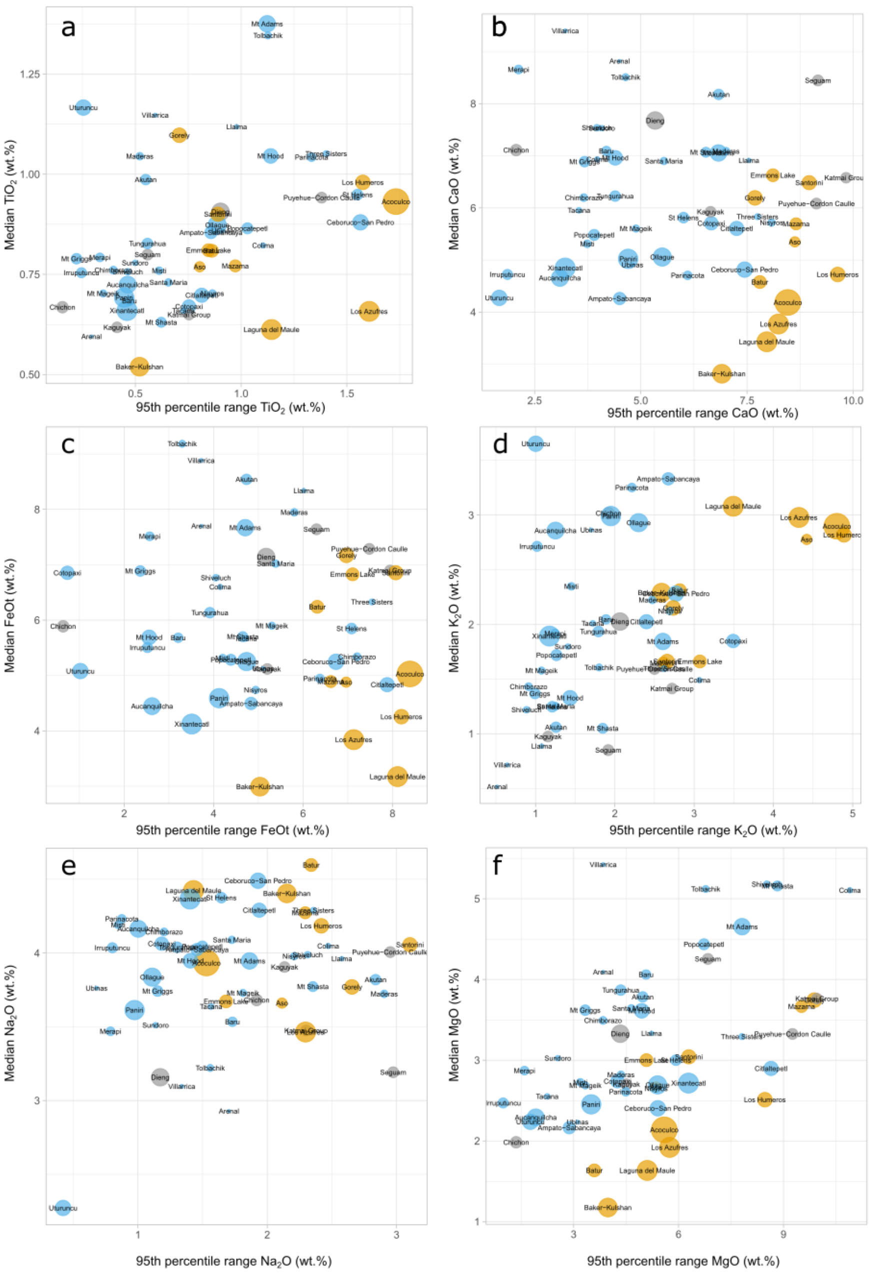

Fig. S2: Median major element oxides in volcanic bulk rock analyses plotted versus the 95th percentile range of the respective oxide in weight percent. Volcanic systems that have produced caldera-forming eruptions in the past (orange symbols) are best separated from systems that have not produced such eruptions yet (blue, grey colours) in major element oxides that show relatively uniform variation in igneous differentiation sequences. Symbol size reflects the total lifespan for each of the volcanic systems.

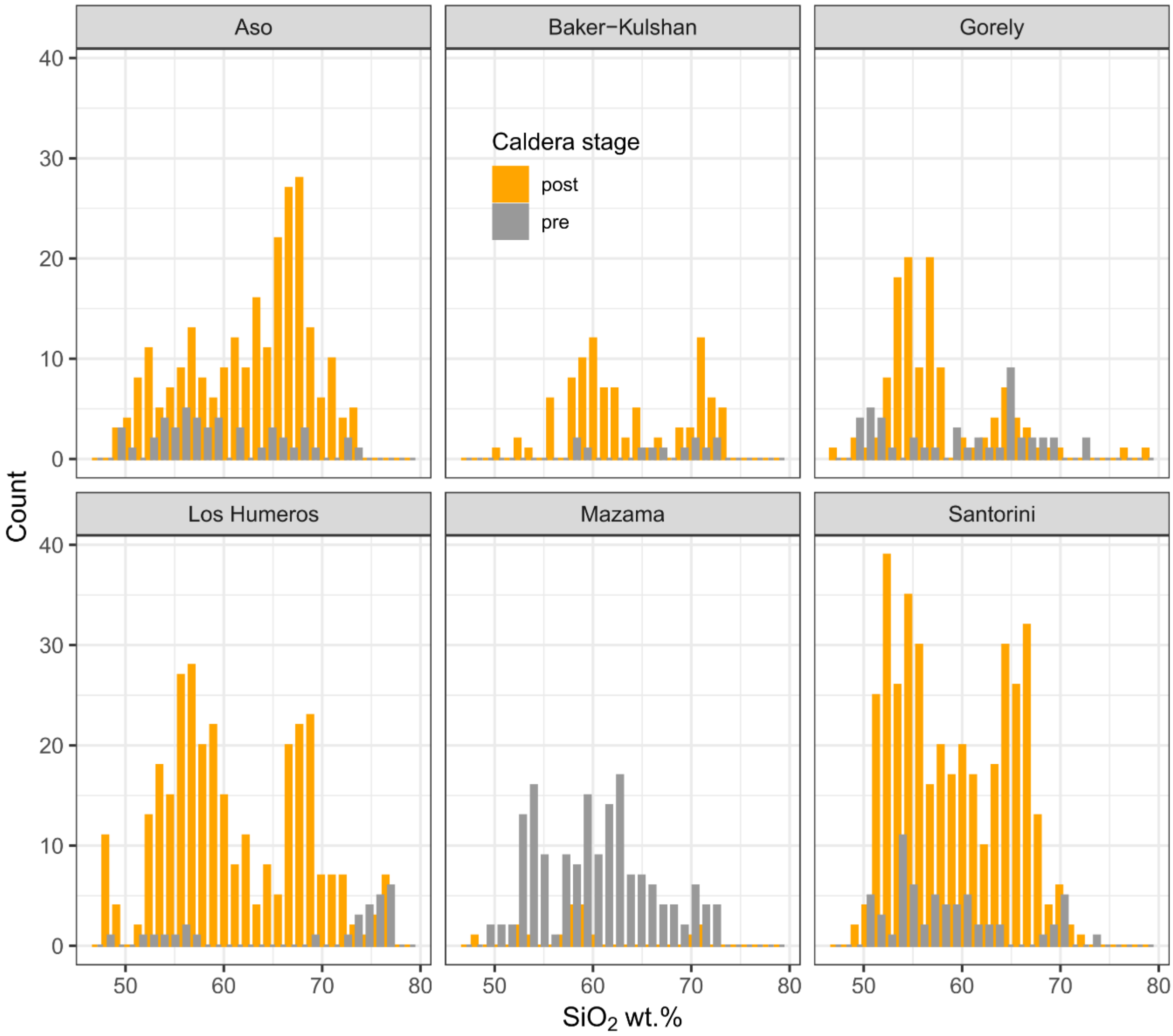

Fig. S3: Histograms of bulk-rock SiO_2 contents (wt.%) for caldera volcanoes. Grey bars show volcanic rocks of the pre-caldera stage before the first caldera-forming eruption of the system, while orange bars indicate the compositional diversity of syn- and post-caldera stages.

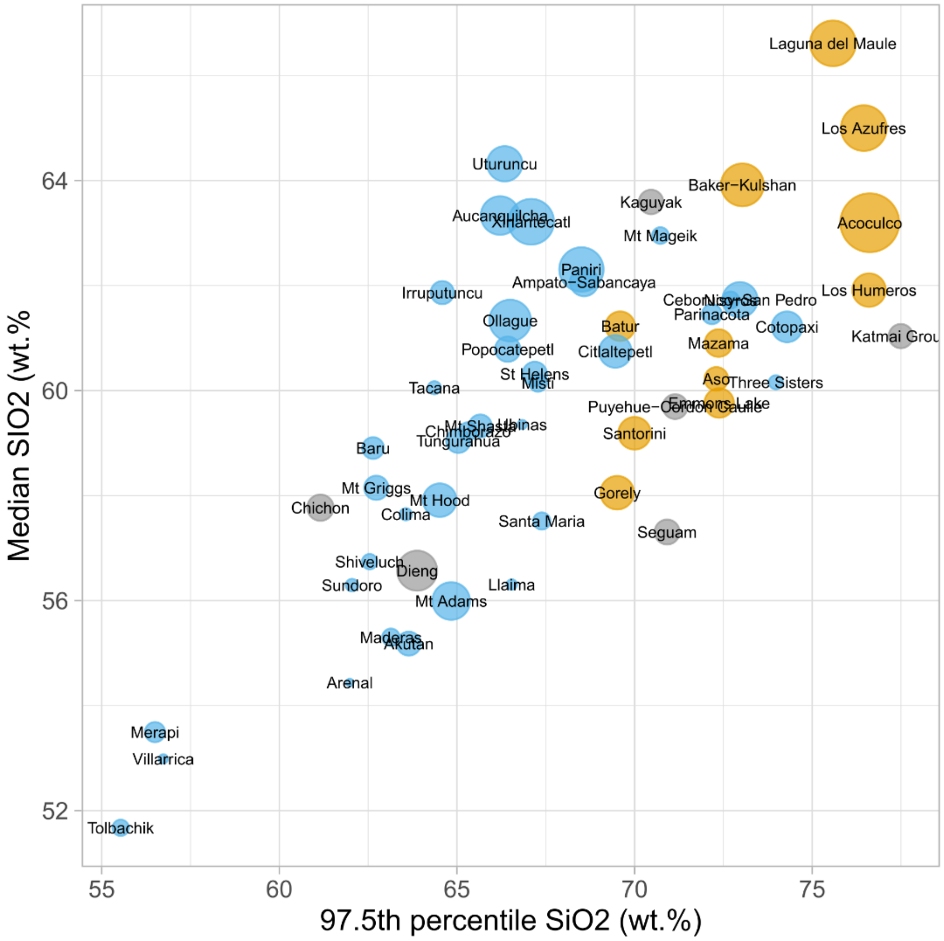

Fig. S4: Median of SiO_2 (wt.%) in volcanic bulk rock analyses plotted versus the 97.5th percentile of SiO_2 contents. Colour coding reflects volcano type: calderas (orange), stratovolcanoes (blue), complexes (grey). Symbol size corresponds to the total duration of activity for each volcano.

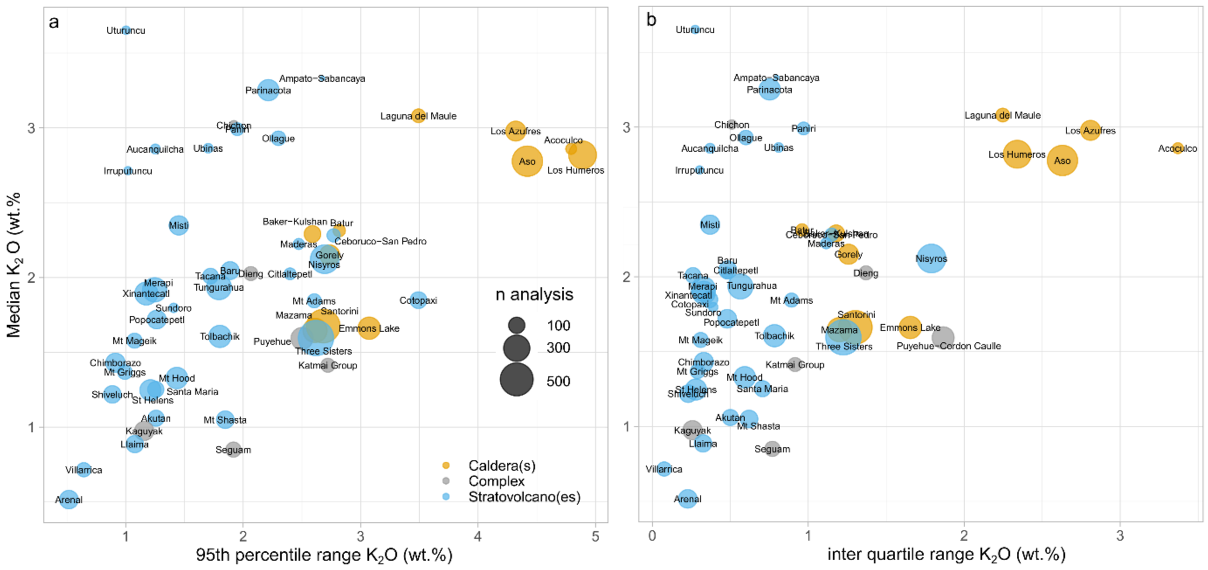

Fig. S5: Median of K_2O (wt.%) in volcanic bulk rock analyses plotted versus the 95th percentile range of K_2O contents a) and against the interquartile range in b). Colour coding reflects volcano type: Calderas are shown in orange, stratovolcanoes in blue, and complexes in grey. Symbol size reflects the number of analysis for each of the volcanic systems.

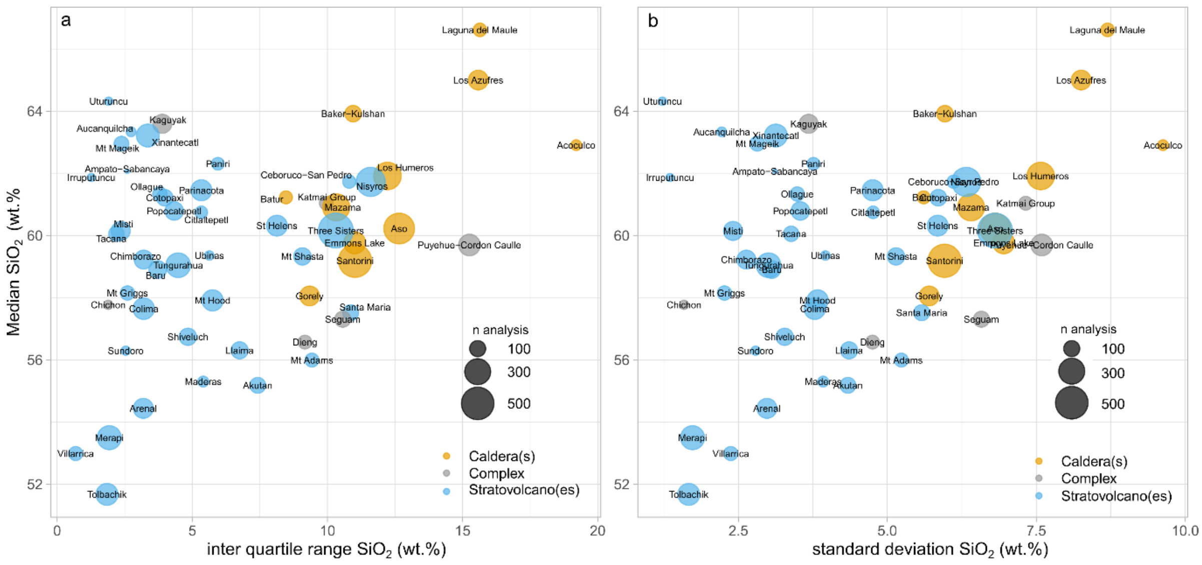

Fig. S6: a) Inter quartile range and b) standard deviation of erupted SiO_2 bulk rock contents (wt.%) from compiled data plotted versus median of SiO_2 content. Symbol size reflects the number of bulk-rock analysis for each volcanic system. Colour coding reflects volcano type.

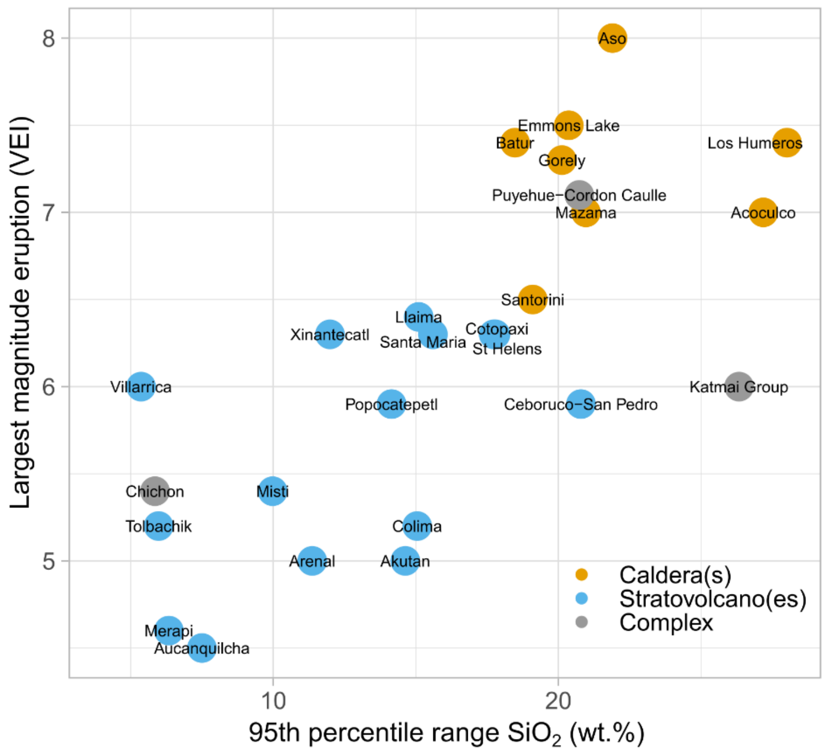

Fig. S7: Range (95th percentile) of SiO_2 contents in weight percent plotted versus the largest eruption magnitude. Eruption magnitudes were compiled from the LAMEVE database (Crosweller et al., 2012) and literature search. Orange colours indicate calderas, stratovolcanoes are shown in blue and complexes in grey.

NOTES ON PRE- / POST-CALDERA DATA COMPILATION

Acoculco. The Acoculco caldera complex in the Eastern Trans Mexican Volcanic Belt was formed around 2.7 Ma by an andesitic ignimbrite. Pre-caldera volcanism (3.0-3.9 Ma) associated with the complex is largely unconstrained. Available bulk-rock compositions of this phase (n=2) are andesitic to dacitic (Avellán et al. 2020).

Aso. The multi-cyclic Asosan caldera in Kyushu (Japan) formed 266±14 ka ago by the evacuation of the Aso-1 ignimbrite. Pre-caldera volcanism in the Aso area has been extensively studied in the caldera walls through radiometric dating, petrography, and whole rock geochemistry (Miyoshi et al. 2009). The pre-caldera eruptive products are mostly younger than 1 Ma and comprise an age range between 2.2 and 0.43 Ma. Like the post-caldera rocks, bulk-rock composition of this early phase span the entire range from basaltic to rhyolitic.

Baker-Kulshan. The Kulshan caldera was formed by an ignimbrite around 1.15 Ma ago with minimum volume of 50 km^3 that is only preserved as intra-caldera deposit likely due to extensive glacial erosion in the area. Pre-caldera rocks have been constrained to range from andesite to rhyodacite, while basaltic rocks have to date only be described in the post-caldera phase, which includes Mt. Baker volcano (Hildreth et al. 2003).

Batur. Collapse of the first Batur caldera on Bali (Indonesia) is associated with the 29.3 ka dacitic Ubud Ignimbrite. While the post-caldera petrology and geochemistry of the Batur volcanic complex has been studied in detail, to our knowledge only few whole-rock compositions are available for the pre-caldera phase (n = 2; Reubi and Nicholls 2004, 2005).

Emmons Lake. The Emmons Lake Volcanic Center (Aleutian arc) is comprised of multiple nested calderas that formed between 27 and 420 ka, producing extensive ashflow tuff deposits (Mangan et al. 2009). While much geochemical work has been carried out on the younger eruptive products, the compositional spread of the pre-caldera episode is unknown.

Gorely. Caldera-forming eruptions at Gorely volcano on the Kamchatka peninsula (Russia) took place between 361 and 38 ka. The pre-caldera stage of the volcano (700-361 ka), termed ‘Old Gorely’ has been studied in some detail and spans, like the post-caldera stage, the entire range of geochemical compositions from basalt to rhyolite (Seligman et al. 2014; Gavrilenko et al. 2016).

Laguna del Maule. Two Pleistocene ignimbrites have been described and dated in the Laguna del Maule area: the 1.5 Ma Sin Puerto dacite and the 990 ka Bobadilla rhyodacite (Hildreth et al. 2010; Andersen et al. 2017). However, while there is geological evidence that at least the latter of these eruptions resulted in caldera formation, the geochemical diversity of pre-caldera volcanic rocks at Laguna del Maule is at the time of writing unknown.

Los Azufres. A remnant wall of the Los Azufres caldera has been described in the Santa Inés range on the northern edge of the volcanic field but may pre-date the currently active volcano. If Los Azufres represents a resurgent caldera or a different structure remains debated (Arce et al. 2012). An analysis of pre- and post-caldera volcano geochemistry is at present not possible.

Los Humeros. The Los Humeros caldera (Trans Mexican Volcanic Belt) was formed by the Xaltipan ignimbrite around 164 ka based on recent Ar-geochronology. Pre-caldera volcanism is preserved just outside the caldera rim as rhyolitic domes to the west and north (map units Qr3 and Qr4 – age <700 ka), as well as basaltic centres dated around 190 ka to the south and north of the caldera structure (map units Qb2). Mafic and silicic pre-caldera eruptions took place in similar distance to the later caldera fault. The Pliocene basaltic to andesitic Teziutlán lava flows (1.46 – 2.61 Ma) were not included in the compilation of pre-caldera rocks at Los Humeros but are not unlikely to be part of the same magmatic system given typical lifetimes of caldera volcanoes of several Ma, the proximity of surface outcrops within about 5 km to the caldera fault, and a thick layer of this unit beneath the Xaltipan ignimbrite (Carrasco-Núñez et al. 2018).

Mazama. The Mazama-Crater Lake caldera was formed relatively recently in a 'cataclysmic' eruption 7.7 ka ago that represents the first caldera-forming eruption of the volcano. Mazama has a long history of >550 ka, which is exceptionally well constrained both in terms of the geochemistry of the eruptive products and eruption volumes (Bacon and Lanphere, 2006). The large range of magma diversity was already produced in the early history of the volcano. Early eruptive products of the Mazama-Crater Lake complex are comprised of rhyodacitic to dacitic lava domes dated at ~460-400 ka and termed 'pre-Mazama' to distinguish them from the later andesitic-dacitic 'Mazama stratovolcano'. Mafic eruptions of basaltic and basaltic andesite magmas occurred over the entire history of the volcanic complex and while termed mostly 'regional volcanism', vent locations of these eruptions are within <10 km from the present-day crater rim.

Santorini. Steep unconformities in the cliffs of Santorini (Aegean arc, Greece) record evidence for at least three caldera-collapse events. The earliest geological records of caldera-formation date back to ~172 ka associated with the 'Lower Pumice 2' eruption. If earlier major explosive eruptions (Lower Pumice 1, Cape Therma 1-3) have produced calderas as well is unconstrained. To be most conservative, the pre-caldera compilation for Santorini consists of the Akrotiri lavas (650-550 ka) and the Peristeria stratovolcano (550-450 ka), which comprise the earliest phase of constrained activity of the volcano. Eruptive products of these phases are rhyodacitic to basaltic in composition (Druitt et al. 1999).

Table S1: Compilation of pre- and post-caldera bulk-rock magma diversity.

Caldera	Location	Bulk rock analysis in compilation n pre-caldera	n post-caldera	pre-caldera chemistry constrained	First Caldera-forming eruption (age)	Key reference
Acoculco	Mexico	2	39	no	Acoculco andesitic ignimbrite (2.7 Ma)	Avellán et al. 2020
Aso	Japan	46	247	yes	Aso-1 ignimbrite (266 ka)	Miyoshi et al. 2009
Baker-Kulshan	US	12	93	yes	Swift creek ignimbrite (1.15 Ma)	Hildreth et al. 2003
Batur	Indonesia	4	42	no	Ubud Ignimbrite (29.3 ka)	Reubi and Nicholls 2004, 2005
Emmons Lake	Alaska	0	250	no	not named (~420 ka?)	Mangan et al. 2009
Gorely	Kamchatka	45	118	yes	Gorely ignimbrite (361 ka)	Seligman et al. 2014; Gavrilenko et al., 2016
Laguna del Maule	Chile	0	68	no	Sin Puerto ignimbrite? (1.5 Ma)	Hildreth et al. 2010; Andersen et al. 2017
Los Azufres	Mexico	0	159	no	unknown	Arce et al. 2012
Los Humeros	Mexico	18	211	yes	Xaltipan ignimbrite (164 ka)	Carrasco-Núñez et al. 2018
Mazama	US	161	17	yes	Mazama climactic eruption (7.7 ka)	Bacon and Lanphere, 2006
Santorini	Greece	61	434	yes	Lower Pumice 2 (172 ka)	Druitt et al. 1999

Supplementary References

Arce, J. L., Macías, J. L., Rangel, E., Layer, P., Garduño-Monroy, V. H., Saucedo, R., García, F., Castro, R. & Pérez-Esquivias, H. (2012). Late Pleistocene rhyolitic explosive volcanism at Los Azufres Volcanic Field, central Mexico. The Southern Cordillera and Beyond. Geological Society of America, 45–82

Avellán, D. R., Macías, J. L., Layer, P. W., Cisneros, G., Sánchez-Núñez, J. M., Gómez-Vasconcelos, M. G., ... & Benowitz, J. (2019). Geology of the late Pliocene–Pleistocene Acoculco caldera complex, eastern trans-Mexican volcanic belt (México). *Journal of Maps*, *15*(2), 8-18.

Bacon, C. R., & Lanphere, M. A. (2006). Eruptive history and geochronology of Mount Mazama and the Crater Lake region, Oregon. *Geological Society of America Bulletin*, *118*(11-12), 1331-1359.

Carrasco-Núñez, G., Bernal, J. P., Davila, P., Jicha, B., Giordano, G., & Hernández, J. (2018). Reappraisal of Los Humeros volcanic complex by new U/Th zircon and 40Ar/39Ar dating: Implications for greater geothermal potential. *Geochemistry, Geophysics, Geosystems*, *19*(1), 132-149.

Crosweller, H. S., Arora, B., Brown, S. K., Cottrell, E., Deligne, N. I., Guerrero, N. O., ... & Venzke, E. (2012). Global database on large magnitude explosive volcanic eruptions (LaMEVE). *Journal of Applied Volcanology*, *1*(1), 1-13.

Gavrilenko, M., Ozerov, A., Kyle, P. R., Carr, M. J., Nikulin, A., Vidito, C., & Danyushevsky, L. (2016). Abrupt transition from fractional crystallization to magma mixing at Gorely volcano (Kamchatka) after caldera collapse. *Bulletin of Volcanology*, *78*(7), 1-28.

Hildreth, W., Fierstein, J., & Lanphere, M. (2003). Eruptive history and geochronology of the Mount Baker volcanic field, Washington. *Geological Society of America Bulletin*, *115*(6), 729-764.

Mangan, M., Miller, T., Waythomas, C., Trusdell, F., Calvert, A., & Layer, P. (2009). Diverse lavas from closely spaced volcanoes drawing from a common parent: Emmons Lake Volcanic Center, Eastern Aleutian Arc. *Earth and Planetary Science Letters*, *287*(3-4), 363-372.

Miyoshi, M., Furukawa, K., Shinmura, T., Shimono, M., & Hasenaka, T. (2009). Petrography and whole-rock geochemistry of pre-Aso lavas from the caldera wall of Aso volcano, central Kyushu. *Journal of the Geological Society of Japan*, *115*, 672-687.

Reubi, O., & Nicholls, I. A. (2004). Magmatic evolution at Batur volcanic field, Bali, Indonesia: petrological evidence for polybaric fractional crystallization and implications for caldera-forming eruptions. *Journal of Volcanology and Geothermal Research*, *138*(3-4), 345-369.

Reubi, O., & Nicholls, I. A. (2005). Structure and dynamics of a silicic magmatic system associated with caldera-forming eruptions at Batur volcanic field, Bali, Indonesia. *Journal of Petrology*, *46*(7), 1367-1391.

Seligman, A., Bindeman, I., Jicha, B., Ellis, B., Ponomareva, V., & Leonov, V. (2014). Multi-cyclic and isotopically diverse silicic magma generation in an arc volcano: Gorely eruptive center, Kamchatka, Russia. *Journal of Petrology*, *55*(8), 1561-1594.
